# Establishing an Efficient Way to Utilize the Drought Resistance Germplasm Population in Wheat

**DOI:** 10.1155/2013/489583

**Published:** 2013-05-07

**Authors:** Jiancheng Wang, Yajing Guan, Yang Wang, Liwei Zhu, Qitian Wang, Qijuan Hu, Jin Hu

**Affiliations:** ^1^Seed Science Center, College of Agriculture and Biotechnology, Zhejiang University, Hangzhou 310058, China; ^2^Shandong Crop Germplasm Center, Shandong Academy of Agricultural Sciences, Jinan 250100, China

## Abstract

Drought resistance breeding provides a hopeful way to improve yield and quality of wheat in arid and semiarid regions. Constructing core collection is an efficient way to evaluate and utilize drought-resistant germplasm resources in wheat. In the present research, 1,683 wheat varieties were divided into five germplasm groups (high resistant, HR; resistant, *R*; moderate resistant, MR; susceptible, *S*; and high susceptible, HS). The least distance stepwise sampling (LDSS) method was adopted to select core accessions. Six commonly used genetic distances (Euclidean distance, Euclid; Standardized Euclidean distance, Seuclid; Mahalanobis distance, Mahal; Manhattan distance, Manhat; Cosine distance, Cosine; and Correlation distance, Correlation) were used to assess genetic distances among accessions. Unweighted pair-group average (UPGMA) method was used to perform hierarchical cluster analysis. Coincidence rate of range (CR) and variable rate of coefficient of variation (VR) were adopted to evaluate the representativeness of the core collection. A method for selecting the ideal constructing strategy was suggested in the present research. A wheat core collection for the drought resistance breeding programs was constructed by the strategy selected in the present research. The principal component analysis showed that the genetic diversity was well preserved in that core collection.

## 1. Introduction

Drought is probably the most important abiotic stress that limits plant growth [1]. Drought stress is one of the most serious environmental factors that can severely limit the yield and quality of agricultural crops [2]. With global climate change, the lack of water for agronomic purposes will become the major problem for crop production [3]. In agronomical point-of-view, drought stress is a situation in which lack of water exceeds the capacity of plants which leads to the growth prevention. Thus, improving the drought tolerance is a major adaptation strategy for plant production in arid and semiarid regions [4]. In drought prone environments, crop drought resistance is a major factor in the stabilization of crop performance. Drought resistance is now considered by both breeders and molecular biologists as a valid breeding target.

Wheat (*Triticum aestivum* L.) is one of the most important cereals in the world. Drought stress may reduce all yield components in wheat [5]. Drought is the major factor limiting wheat growth and productivity in many regions of the world, and the changing global climate is making the situation more serious [6, 7]. Developing high-yielding wheat cultivars under drought conditions in arid and semiarid regions is an important objective of breeding programs [5]. Although great efforts have been made in wheat drought resistance breeding, the decrease in agricultural productivity induced by drought stress still remains unsolved [8]. One reason is that the numerous germplasm resources were not effectively utilized in wheat breeding programs. However, with continuous collection of germplasm resources, the size of populations has been becoming bigger and bigger, which hindered the evaluation and utilization of the wheat germplasm resources.

Core collections provide an efficient way to evaluate and utilize germplasm resources. A core collection is a representative sample of the whole collection which has minimum repetitiveness and maximum genetic diversity of a plant species [9]. The core collection serves as a working collection to be evaluated and utilized preferentially [10–13]. In this way, it is possible to preserve most of the genes in large germplasm populations using a small sample. Thus, the objectives of this research were (1) to investigate the ideal constructing strategy on wheat core collection based on data of agronomic traits combining drought resistance information and (2) to construct such a wheat core collection for the drought resistance breeding programs.

## 2. Materials and Methods

### 2.1. Materials

Wheat varieties were introduced from abroad. The drought resistance level combining four yield traits (plant height, spike length, grain numbers per spike, and 1000-grain weight) and four quality traits (crude protein content, lysine content, sedimentation, and hardness) of 1,683 varieties have been investigated. All data were downloaded from “Chinese Crop Germplasm Resources Information System” (http://icgr.caas.net.cn/). 

### 2.2. Core Collection Construction

**Figure 1:**
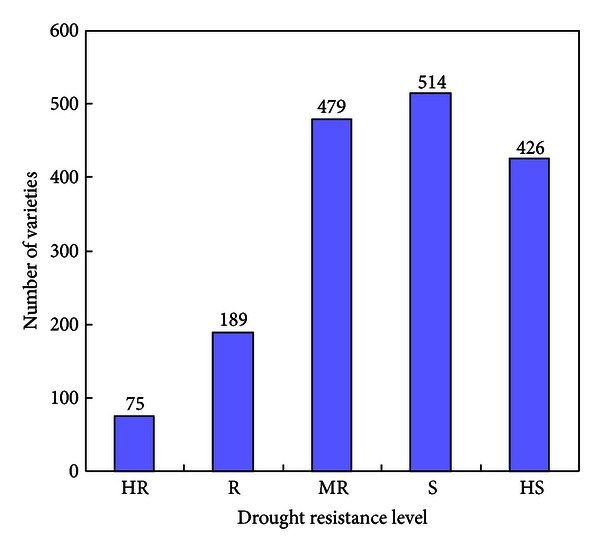
The distribution of 1,683 wheat varieties: HR: high resistant; *R*: resistant; MR: moderate resistant; *S*: susceptible; HS: high susceptible.

According to the drought resistance level, all 1,683 wheat varieties were divided into five germplasm groups (high resistant, HR; resistant, *R*; moderate resistant, MR; susceptible, *S*; and high susceptible, HS). The distribution of varieties was shown in Figure 1. The procedure for core collection construction was conducted by two steps. First, subcore collections were selected from each germplasm groups. Second, all the sub-core collections were combined together to construct a core collection.

The least distance stepwise sampling (LDSS) method [14] was adopted to construct sub-core collections from germplasm groups. The procedure was as follows. (1) The genetic distances among accessions were calculated, and accessions were classified by hierarchical cluster analysis based on their genetic distance. (2) One accession from a subgroup with the least distance was randomly removed, and another accession of the subgroup was sampled. (3) The genetic distances among the remaining accessions were calculated, and the sampling was repeated in the same way. The stepwise samplings were performed until the percentage of the remaining accessions reached the desired one. This method performs sampling based on the subgroup with the least genetic distance, which can efficiently eliminate redundant accessions and ignore the effect of the cluster methods [15].

### 2.3. Genetic Distances and Evaluating Parameters

Six commonly used genetic distances (Euclidean distance, Euclid; Standardized Euclidean distance, Seuclid; Mahalanobis distance, Mahal; Manhattan distance, Manhat; Cosine distance, Cosine; and Correlation distance, Correlation) were used to assess genetic distances among accessions. Unweighted pair-group average (UPGMA) method was used for performing hierarchical cluster analysis [15].

Coincidence rate of range (CR) and variable rate of coefficient of variation (VR) [16, 17] were adopted to evaluate the representativeness of core collection. Those four parameters were formulated as follows: CR = (1/*n*)∑_*i*=1_^*n*^(*R*_*C*(*i*)_/*R*_*I*(*i*)_) × 100, where *R*_*C*(*i*)_ is the range of the *i*th trait in the core collection; *R*_*I*(*i*)_ is the range of the corresponding trait in the initial collection; *n *and is total number of traits, VR = (1/*n*)∑_*i*=1_^*n*^(CV_*C*(*i*)_/CV_*I*(*i*)_) × 100, where CV_*C*(*i*)_ is the coefficient of variation of the *i*th trait in the core collection; CV_*I*(*i*)_ is the coefficient of variation of the corresponding trait in the initial collection; *n* is total number of traits.

### 2.4. Data Analysis

The genetic distances calculation, the LDSS procedures, and the evaluating parameters calculation were performed using computer code programmed by the authors based on MATLAB software (version 6.5) [18].

## 3. Results

### 3.1. The Assessment of Subcore Collections Constructed by Different Genetic Distances

Subcore collections were constructed by different genetic distances at the sampling percentage of 10%, 20%, and 30% (Table 1). In any germplasm group, CR and VR of sub-core collections constructed by the genetic distance of Cosine and Correl were much lower than of those constructed by the other four genetic distances at the three sampling percentages (Table 1). In HR group, sub-core collections constructed by Manhat had larger CR and VR than those constructed by Euclid, Seuclid, and Mahal at the three sampling percentages (Table 1). In *R* group, sub-core collections constructed by Euclid had the largest CR at the sampling percentage of 10% and 30%, and those constructed by Manhat had the largest VR at the sampling percentage of 10% and 20% (Table 1). In MR group, sub-core collections constructed by Mahal had the largest CR at the sampling percentage of 20% and 30%, and those constructed by Seuclid had the largest VR at the sampling percentage of 10% and 20%, but similar VR than that constructed by Euclid at the sampling percentage of 30% (Table 1). In S group, sub-core collections constructed by Seuclid had the largest CR at the three sampling percentage, while there was no significant pattern in VR (Table 1). In HS group, sub-core collections constructed by Euclid had the largest CR at the sampling percentage of 20% and 30%, and those constructed by Mahal had the largest VR at the sampling percentage of 10% and 30% (Table 1). Synthesizing the results above, five ideal combinations for sub-core collection were selected: HR-Manhat, *R*-Euclid, MR-Mahal, *S*-Seuclid, and HS-Euclid.

### 3.2. Selection of the Optimal Sampling Percentage

**Figure 2:**
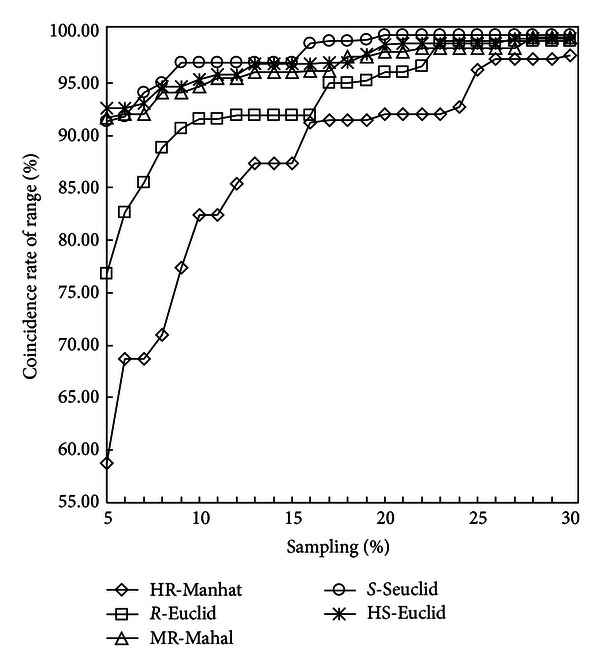
The coincidence rate of the range (CR) of subcore collections constructed by five combinations with the sampling percentage increasing from 5% to 30%. HR-Manhat, high resistant group combining Manhattan distance; *R*-Euclid, resistant group combining Euclidean distance; MR-Mahal, moderate resistant group combining Mahalanobis distance; *S*-Seuclid, susceptible group combining Standardized Euclidean distance; the HS-Euclid, high susceptible group combining Euclidean distance.

In each germplasm group, sub-core collections were constructed based on the selected genetic distance with the sampling percentage increasing from 5% to 30%. The value of CR of each sub-core collection was calculated. Thus, 26 CRs were calculated in each group. The constructing results of the five groups were summarized in Figure 2. In each group, the CR showed logarithmic changing. The CR increased drastically when the sampling percentage was small. With the sampling percentage increasing, CR increased steady (Figure 2). The rangeability in the group of HR and *R* was larger than that in the groups of MR, *S*, and HS (Figure 2).

Each curve of in Figure 2 was treated by curve fitting analysis, and the results were summarized in Table 2. The equations showed logarithmic form, and the coefficient of determination of fitted equations (*R* ^2^) of each equation was larger than 0.9 (Table 2). Based on the equations, the optimal sampling percentage was calculated by setting the value of CR (%) to 95.00 (Table 2).

### 3.3. Validation of the Ideal Constructing Strategy

**Figure 3:**
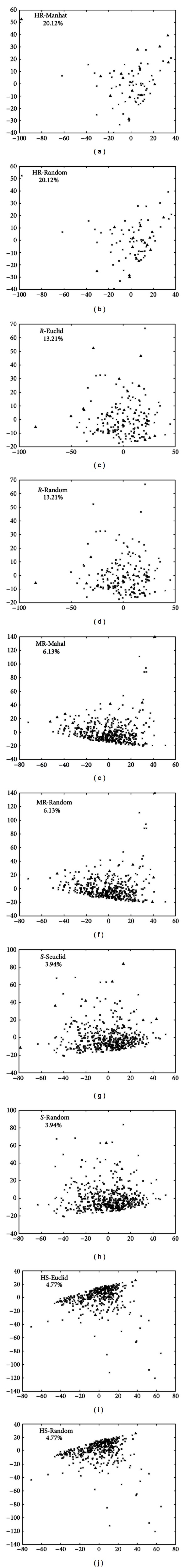
Principal component plots of core accessions and reserve accessions in the sampling percentage. The upward pointing triangles represented the core accessions; the crosses represented the reserved accessions. The left column showed plots for subcore collection constructed by LDSS method based on the selected genetic distance and sampling percentage; the right column showed plots for sub-core collection constructed by complete random selection based on the same sampling percentage. HR-Manhat, high resistant group combining Manhattan distance; *R*-Euclid, resistant group combining Euclidean distance; MR-Mahal, moderate resistant group combining Mahalanobis distance; *S*-Seuclid, susceptible group combining Standardized Euclidean distance; and HS-Euclid, high susceptible group combining Euclidean distance.

The principal component analysis was adopted to validate sub-core collections constructed by the ideal strategy selected by the present research. Principal component plots of core accessions and reserve accessions in each germplasm group were drown in Figure 3. The total genetic variation percentage of the first two principal components was 71.51% in HR group, 67.67% in *R* group, 66.90% in MR group, 68.45% in *S* group, and 71.83% in HS group. At the same sampling percentage, compared to the sub-core collections constructed by complete random selection, the core accessions selected by the present strategy showed more symmetrical distribution in the whole germplasm group, and most extreme accessions were selected (Figure 3).

## 4. Discussion

Core collection has been studied for about twenty years [19, 20]. A valid core collection provides a high-efficient way to assess genetic diversity or to find beneficial genes [21–24]. Most core collection researches focused on finding efficient ways in sub-core collection selection [25–27]. However, there is not a widely accepted strategy for constructing sub-core collection up to now. One common approach for constructing a core collection is splitting the whole germplasm population into several groups, then, selecting representative core accessions from each group to form sub-core collections, and combining all sub-core collections to form the final core collection [16, 28]. The present research divided the whole wheat germplasm population into five groups based on drought resistance level. The results showed that the distribution pattern of accessions was various in different germplasm group, which might lead to different suitable strategy for sub-core collection construction. Therefore, different germplasm group required different constructing strategy, and it is needlessly to try to find a widely accepted constructing strategy.

 The representativeness is the most important character for a core collection. The VR represents the difference of variance between core collection and the initial collection. The value of VR is affected greatly by the number of accessions in the core collection. In core collection construction based on a valid strategy, with the sampling percentage increasing, the variance decreased and the mean almost keeps unchanging, which led to the decrease of VR. However, at the same sampling percentage, bigger VR means more variation preserved in core collection. The CR shows the extent of preservation of the trait scope in a core collection. The value of CR is not affected greatly by the number of accessions. In the present research, the CR showed sensitivity to the representativeness of a sub-core collection. The CR has been reported to be an important parameter for the evaluation of the representativeness of the core collections [9, 29, 30]. Based on the above analysis, the ideal genetic distance for different group was determined first by CR, then by VR. Moreover, a genetic distance that could make higher CR at low sampling percentage might be more valid than others.

In the present research, data of eight agronomic traits in 1,683 wheat varieties were downloaded from public database of “Chinese Crop Germplasm Resources Information System.” Such a big number of wheat germplasm might not be planted within one area or one year. Therefore, the upper data might not be collected based on the same cultivating standards, which might affect the precision of the final core collection. However, there were more than one agronomic trait used in the present research. Data of eight agronomic traits were used to calculate CR and VR. The two evaluating parameters reflected the mean representativeness of the eight agronomic traits in the core collection, which reduced the error mentioned above. A wheat core collection for the drought resistance breeding programs was constructed by the strategy selected in the present research based on the upper dataset. Table 2 showed the optimal genetic distance and the relative optimal sampling percentage for sub-core collection in each germplasm group. Therefore, the whole core collection was constructed by combining all sub-core collections. The principal component analysis showed that the genetic diversity was well preserved in that core collection. The method for the ideal constructing strategy selection suggested in the present research is also valuable in other crop's core collection construction.

## Figures and Tables

**Table 1:** The values of CR and VR of different subcore collection constructed by six genetic distances at the sampling percentage of 10%, 20%, and 30%.

Parameter	DRL^(a)^	SP^(b)^	Genetic distance					
			Euclid	Seuclid	Mahal	Manhat	Cosine	Correl
CR (%)		10%	76.17	87.16	80.48	82.36	51.34	52.54
	HR	20%	89.55	91.96	88.77	91.98	71.08	70.14
		30%	95.78	94.17	93.76	97.59	77.57	80.69
		10%	91.51	81.60	84.16	89.24	60.62	59.34
	*R*	20%	95.98	86.40	92.55	96.09	73.60	65.00
		30%	98.93	96.11	93.64	96.88	81.25	72.20
		10%	94.20	92.61	94.58	95.37	58.36	70.18
	MR	20%	97.41	96.27	98.00	97.10	66.23	78.06
		30%	98.37	97.35	99.31	97.11	75.63	86.26
		10%	96.46	96.91	96.05	95.24	69.75	65.32
	*S*	20%	97.81	99.58	98.92	99.46	75.42	72.74
		30%	98.04	99.58	99.35	99.49	82.47	77.19
		10%	95.33	93.92	95.88	93.88	61.97	63.68
	HS	20%	98.65	96.43	97.30	96.81	80.81	73.24
		30%	99.20	97.82	97.82	99.20	82.20	82.80

VR (%)		10%	122.67	127.61	124.51	131.10	85.27	79.12
	HR	20%	122.60	120.78	119.30	125.19	92.14	90.12
		30%	117.73	119.07	113.57	122.80	91.74	91.82
		10%	129.66	118.85	118.24	132.61	90.21	82.94
	*R*	20%	119.06	115.52	117.51	120.73	88.99	82.92
		30%	112.89	114.32	113.78	113.18	90.82	88.82
		10%	128.26	128.60	126.81	128.51	80.95	90.70
	MR	20%	119.56	122.15	120.58	120.39	85.01	92.93
		30%	116.79	116.78	113.45	115.29	89.06	92.51
		10%	130.14	129.53	126.03	127.10	90.40	85.16
	*S*	20%	120.64	120.67	119.72	121.22	88.94	88.29
		30%	115.44	115.40	115.53	114.36	92.36	90.24
		10%	125.25	128.97	133.16	124.54	89.94	90.06
	HS	20%	122.00	122.77	122.13	120.35	92.00	91.91
		30%	117.09	116.11	117.42	115.94	87.68	93.25

^(a)^DRL: drought resistance level (HR: high resistant; *R*: resistant; MR: moderate resistant; *S*: susceptible; and HS: high susceptible).

^(b)^SP: sampling percentage.

**Table 2:** The logarithmic equations on the CR's value responded to the sampling percentage in five combinations of subcore collection construction. The optimal sampling percentage was calculated by the equation when the value of CR (%) was set to 95.00.

Combination^(a)^	Equation^(b)^	*R* ^2(c)^	Optimal sampling percentage (%)
HR-Manhat	*y* = 12.19ln(*x*) + 58.41	0.9732	20.12
*R*-Euclid	*y* = 6.52ln(*x*) + 78.17	0.9616	13.21
MR-Mahal	*y* = 2.67ln(*x*) + 90.16	0.9451	6.13
*S*-Seuclid	*y* = 2.80ln(*x*) + 91.16	0.9392	3.94
HS-Euclid	*y* = 2.47ln(*x*) + 91.14	0.9498	4.77

^(a)^HR-Manhat: high resistant group combining Manhattan distance; *R*-Euclid: resistant group combining Euclidean distance; MR-Mahal: moderate resistant group combining Mahalanobis distance; *S*-Seuclid: susceptible group combining Standardized Euclidean distance; and HS-Euclid: high susceptible group combining Euclidean distance.

^(b)^ *x*: the sampling percentage (%); *y*: the value of CR (%).

^(c)^ *R* ^2^: coefficient of determination of fitted equations.
